# A retrospective analysis of usefulness of impulse oscillometry system in the treatment of asthma

**DOI:** 10.1186/s12931-020-01494-x

**Published:** 2020-08-31

**Authors:** Hiroyuki Sugawara, Atsushi Saito, Saori Yokoyama, Kazunori Tsunematsu, Hirofumi Chiba, Hiroki Takahashi

**Affiliations:** 1 Sugawara Internal Medicine and Respiratory Clinic, Tomakomai, 053-0821 Japan; 2 Department of Respiratory Medicine and Allergology, Sapporo Medical University School of Medicine, S1W16 Chuoku, Sapporo, Hokkaido 060-8543 Japan

**Keywords:** Bronchial asthma, Inhaled corticosteroids, Impulse oscillometry system, Pulmonary function test, Asthma health questionnaire, Asthma control test

## Abstract

**Background:**

Bronchial asthma (BA) has different phenotypes, and it requires a clinically effective subtype classification system. The impulse oscillometry system (IOS) is an emerging technique device used in respiratory functional tests. However, its efficacy has not been validated. Therefore, this study aimed to assess the relationship between BA and the IOS parameters, and the difference in the therapeutic effects of inhaled corticosteroids (ICSs) among the subtype classifications was evaluated using the IOS.

**Methods:**

Of the 245 patients with bronchial asthma who were screened, 108 were enrolled in this study. These patients were divided based on three subtypes according to the IOS result as follows: central predominant type (*n* = 34), peripheral predominant type (*n* = 58), and resistless type (*n* = 16). Then, the following ICSs were randomly prescribed in daily medical care: coarse-particle ICS (fluticasone propionate [FP]), fine-particle ICS (mometasone furoate [MF]), and moderate-particle ICS (budesonide [BUD]). The treatment effects were assessed using the Asthma Health Questionnaire (AHQ) and the Asthma Control Test (ACT) and were compared among the three subtypes.

**Results:**

In the central predominant type, the AHQ score of the MF group was significantly higher than that of the FP group (15.4 vs. 3.6, *p* < 0.01) and the BUD group (15.4 vs. 8.8, *p* < 0.05); the ACT score of the FP group was significantly higher than that of the MF and BUD groups (24.3 vs. 21.7, 22.3, respectively, *p* < 0.05) at 4 weeks after treatment. In the peripheral predominant type, the AHQ score of the FP group was significantly higher than that of the MF group (14.1 vs. 3.4, *p* < 0.05); the ACT score of the FP group was lower than that of the MF and BUD groups (22.8 vs. 24.6, 24.4, respectively, *p* < 0.01) at 4 weeks after treatment.

**Conclusions:**

An association was observed between IOS subtype classification and ICS particle size in terms of therapeutic efficacy in BA. This result indicates that the IOS could be an effective tool in the selection of ICS and the evaluation of the BA phenotype.

## Background

Asthma is one of the most common chronic respiratory diseases affecting 1–18% of the population in different countries, and it is typically characterized by chronic airway inflammation. Moreover, individuals with asthma present with variable expiratory airflow limitation and respiratory symptoms, such as wheezing, shortness of breath, chest tightness, and cough, which vary over time and in intensity [1]. Asthma is a heterogeneous disease, and the cluster of demographic, clinical and/or pathophysiological characteristics are often referred to as asthma phenotypes [2–4]. However, clinical phenotypes or treatment responses are not correlated to pathological features [5]. Thus, further research should be performed to assess the clinical efficacy of a phenotypic classification system for asthma. The impulse oscillometry system (IOS) can be used to assess the parameters of large and small airway functions. Moreover, it is an emerging technique and noninvasive device used for the assessment of respiratory function using the forced oscillation technique, which was first described by Dubois et al. [6–8]. This device uses sound waves to rapidly detect airway changes. These pressure signals can independently quantify the degree of obstruction in the central and peripheral airways. Moreover, ICSs with different particle sizes are commercially available. In asthma, therapeutic effects may differ based on the drug aerosol’s particle size and location of inflammation [9, 10]. Our recent study revealed that a relationship exists between the therapeutic effect based on the particle size of ICSs and the parameters of IOS in CVA patients [11]. Using the IOS, the cough variant asthma (CVA) subtypes were classified as follows: the central, peripheral, and resistless type. Coarse- and fine-particle ICSs are effective for patients with central and peripheral airway resistance, respectively [11]. IOS values are reported to more correlate with clinical symptoms in asthma compared with spirometry as well as CVA, because it is possible to detect subtle airway changes earlier than spirometry [12, 13]. Airway inflammation in BA may extend from the central to the peripheral airways [14]. Thus, several studies have assessed the clinical application of the IOS for the treatment of BA [15, 16].

In this study, we examined the effect of dry powder inhaler (DPI) and excluded pressurized metered dose inhaler (pMDI) device because pMDI and DPI are completely different in terms of the inhalation procedure. For instance, DPI requires a strong inspiratory flow velocity, and breath holding after inhalation appears to be important for pMDI.

In the current study, the clinical role of the IOS in BA was assessed by dividing the participants according to three subtypes with the R20 and R5 − R20 values. Moreover, whether the IOS can be utilized in the diagnosis and therapeutic evaluation of BA was assessed.

## Method

### Participants and treatments

This investigation is a single-center retrospective observational study. Patients who presented with ICS-na*ï*ve BA and visited our clinic for the first time between April 2017 and June 2019 were included in this study. BA was defined as the presence of variable expiratory airflow limitation and history of respiratory symptoms, such as wheezing, shortness of breath, chest tightness, and cough, which vary over time and in intensity. Patients who had respiratory symptoms and detailed history and examination results supporting asthma diagnosis were diagnosed with asthma based on variable airflow limitation, as evidenced by reduced forced expiratory volume in 1 s (FEV1) and FEV1/forced vital capacity (FVC) ratio, and a significant increase in lung function 4 weeks after anti-inflammatory treatment [1]. Patients diagnosed with bronchial asthma were treated according to the Japanese asthma prevention and management guidelines [17]. Three ICSs were used for treatment and were randomly assigned to the patient groups: fluticasone propionate (FP: average particle size = 4.4 um [coarse particle]), mometasone furoate (MF: average particle size = 2.0 um [fine particle]), and budesonide (BUD; average particle size = 2.4 um [moderate particle]). In addition to the ICS therapy, all patients were treated with salmeterol or formoterol (long-acting beta2-agonists) and montelukast (leukotriene receptor antagonists). Therapeutic efficacy was assessed at baseline and 2 and 4 weeks after treatment using the Asthma Health Questionnaire (AHQ)-33-Japan and at baseline and 4 weeks after treatment with the asthma control test (ACT). IOS was performed as a supplementary diagnosis for research purpose. Participants were divided into three groups according to the IOS value after the start of the study. The experimental protocols and the purpose of the research were explained to all participants and informed consent was obtained in the form of opt-out on the web-site. The study was conducted in accordance with the declaration of Helsinki and was approved by the institutional ethical committee of Sapporo Medical Association.

### Measurements of IOS and pulmonary function

IOS measurements were assessed using a commercially available IO device (Master Screen IOS, Jaeger, Germany) according to the manufacturer’s recommendations [8]. The resistance at 5 Hz (R5: indicating total airway resistance), resistance at 20 Hz (R20: representing central airway resistance), difference between R5 and R20 (R5 − R20: index of the small airways), reactance at 5 Hz (X5: relating to compliance), resonant frequency (Fres), and integrated area of low-frequent X (AX) values were evaluated [18–20]. The use of Fres and AX for detecting expiratory flow limitations was proposed.

After the IOS measurement, spirometry was performed by using MasterScreen IOS-Jaeger (Germany). To prevent the occurrence of any negative effects caused by forced expiration on the airway, spirometry was not performed before IOS measurement. The percentage predicted forced vital capacity (%FVC), percentage predicted forced expiratory volume in 1 second (%FEV_1_), FEV_1_/FVC ratio, percentage predicted maximal mid-expiratory flow (%MMEF), and percentage predicted peak expiratory flow (%PEF) were assessed.

The predicted values (PV) of the parameters of IOS were calculated using the equations reported by Vogel et al. [21]. The patients diagnosed with BA were divided based on three subtypes, as shown in Table 1.

**Table 1:** Definition of the subtypes of bronchial asthma classified using the impulse oscillometry. The predicted values of the parameters using the impulse oscillometry system were calculated using the equations reported by Vogel et al

	R20	R5 − R20	(R20 − PV) − ([R5 − R20] − PV)
Central predominant (CP) type	≥100%	< 100%	–
	≥100%	≥100%	≥0
Peripheral predominant (PP) type	< 100%	≥100%	–
	≥100%	≥100%	< 0
Resistless type	< 100%	< 100%	–

### AHQ and ACT

All participants with asthma completed the AHQ at baseline and 2 and 4 weeks after treatment and the ACT at baseline and 4 weeks after treatment.

The Japanese version of the AHQ, which is a disease-specific, health-related quality of life questionnaire, was developed [22, 23]. The clinical validity of the AHQ was evaluated, and it was found to be reliable and effective for discriminative purposes. Thus, it can be used with confidence in clinical research. The AHQ has six subscales (asthmatic symptom, emotion, daily activity, factors that worsened symptoms, social activity, and economics) and comprises 32 items (grades 0–4) and one face scale (grades 1–5). A higher AHQ score reflect a worse health status with respect to these 33 items.

The ACT is a validated, patient-completed measure of asthma control, which comprises five questions used to assess activity limitation, shortness of breath, night-time symptoms, use of rescue medication, and overall rating of asthma control within the last 4 weeks [24]. The questions are scored from 1 (worst) to 5 (best), and the ACT score is obtained by obtaining the sum of the responses, with a maximum best score of 25. An ACT score of 19 indicated the highest area under the ROC curve. Thus, a score ≥ 20 is considered the optimal cutoff point for a well-controlled asthma within the last 4 weeks.

### Measurement of FeNO

Fractional exhaled nitric oxide (FeNO) measurement was performed according to the 2011 recommendations of the American Thoracic Society [25] and prior to spirometry to prevent modifying its values. Niox® VERO (Circassia AB, Sweden), a portable analyzer, was used to assess whether the expiratory flow was maintained at 50 mL/s using acoustic emission signal.

### Statistical analysis

Numeric variables were expressed as means ± standard error of mean. Differences among the groups were assessed with non-repeated analysis of variance with or without the Student–Newman–Keuls test. The differences before and after treatment were compared using paired *t*-test or Wilcoxon signed-rank test. Categorical variables were tested using the chi-square test. A *p* value < 0.05 was considered statistically significant. Microsoft Excel 2007 (Microsoft Corporation, the USA), Excel Statistical Program File (ystat2008.xls, Igakutosho-shuppan Ltd., Tokyo, Japan), and GraphPad Prism v8 software (GraphPad, Inc., San Diego, CA, the USA) were used for data analysis and graph generation. Spearman’s correlation analysis and JMP13.0 (SAS Institute, Cary, NC, the USA) were used to evaluate the coefficients of determination (ρ), residuals, and significance (p) to identify the associations between the pulmonary function test score and IOS index.

## Results

### Selection of participants

**Fig. 1:**
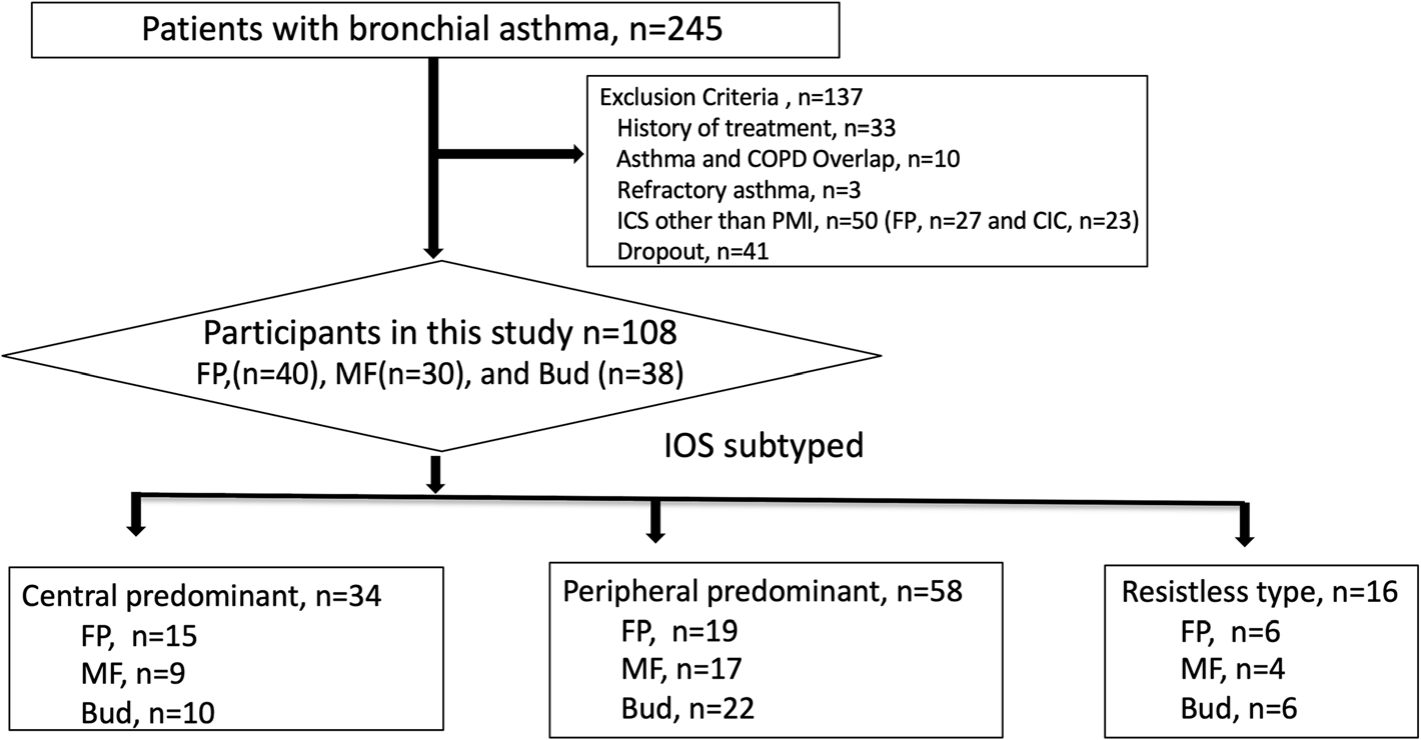
Selection of participants. Of the 245 patients diagnosed with bronchial asthma who visited the clinic between April 2017 and June 2019, 137 were excluded and 108 participated. In total, 34 patients with central predominant type, 58 with peripheral predominant type, and 16 with resistless type were enrolled. The definition of the subtypes of bronchial asthma classified using the impulse oscillometry system is shown in Table 1

Of the 245 patients with bronchial asthma who were screened, 137 were excluded due to a history of treatment (*n* = 33), asthma and COPD overlap (*n* = 10), and refractory asthma (n = 3), use of ICS other than dry powder inhalation (*n* = 50), and dropout (*n* = 41). In total, 108 patients were enrolled in this study. These patients were divided based on three subtypes according to the IOS result, as described in the Methods section: CP type (*n* = 34), PP type (*n* = 58), and resistless type (*n* = 16). In addition, these patients were randomly prescribed with any ICS (FP, MF, or BUD) to compare the therapeutic effects of the drug: 34 patients with the CP type (FP: 15, MF: 9, and BUD: 10), 58 patients with the PP type (FP: 19, MF: 17, and BUD: 22), and 16 patients with the resistless type (FP: 6, MF: 4, and BUD: 6) (Fig. 1).

### Baseline characteristics of the participants

The characteristics of the patients are summarized in Table 2. The number of young participants in the CP group was slightly higher than that in the other groups. No difference was observed among the three groups in terms of sex ratio, duration of disease, smoking rate, BMI, IgE level, number of eosinophils in the peripheral blood, and FeNO. In terms of spirometry parameters, the %FEV1, FEV1/FVC ratio, and %MMEF of the PP type were significantly lower than those of the CP and resistless types. The %PEF of the resistless type was higher than that of the CP and PP types. In addition, the characteristics of the three ICS groups are summarized in Table S1. No statistically significant differences were observed in terms of characteristics among the groups.

**Table 2:** Baseline characteristics of the study participants and the subgroups. Data were presented as mean (standard error of mean) or number (percentage). The differences among the subtypes were assessed using non-repeated analysis of (†P) with the Student–Newman–Keuls test for age, duration of disease, body mass index, IgE level, number of eosinophil in the blood, and spirometric parameter (post-hoc test). The differences in sex and smoking were tested using the chi-square test. *: *p* < 0.05, **: *p* < 0.01 and NS: not significant. CP-PP: between the central predominant type and peripheral predominant type; CP-R: between the central predominant type and resistless type; and PP-R: between the peripheral predominant type and resistless type

	All	Subtypes				Post-hoc test		
		CP	PP	R	P	CP-PP	CP-R	PP-R
n	108	34	58	16				
Characteristics of the patients								
Age	47.9 (1.7)	41.3 (2.7)	52.0 (2.3)	50.0 (4.0)	*	*	NS	NS
Male/female (% of female)	45/64	14/20 (58.8)	19/39 (67.2)	11/5 (31.1)	NS			
Duration of disease (years)	4.9 (0.7)	5.1 (1.3)	5.4 (0.8)	2.4 (0.7)	NS			
Smoker/non-smoker (% of smokers)	68/40	20/14 (58.8)	37/21 (63.8)	11/5 (68.8)	NS			
BMI	25.3 (0.5)	25.6 (0.9)	25.6 (0.6)	23.8 (1.0)	NS			
Total IgE level (IU/mL)	410.0 (60.4)	440.1 (138.8)	389.9 (79.9)	426.7 (87.0)	NS			
Eosinophil in the blood (/μl)	278.4 (18.9)	227.6 (138.9)	306.4 (22.4)	271.4 (36.1)	NS			
FeNO (ppb)	71.0 (5.8)	54.9 (9,7)	72.4 (8.0)	100.0 (13.0)	NS			
Spirometric values								
% FVC	80.9 (1.7)	82.6 (2.9)	77.0 (2.4)	91.0 (2.3)	**	NS	NS	*
% FEV1	67.3 (1.9)	72.4 (3.1)	61.4 (2.7)	77.9 (2.0)	**	*	NS	**
FEV1/FVC ratio	69.4 (1.1)	75.1 (1.9)	65.2 (1.5)	72.8 (2.3)	**	**	NS	*
%MMEF	33.9 (2.0)	43.8 4.0)	26.1 (2.1)	41.3 (4.2)	**	**	NS	**
% PEF	73.1 (2.4)	74.2 (3.5)	66.4 (3.4)	95.3 (4.0)	**	NS	**	**

### Therapeutic evaluation with the AHQ

**Fig. 2:**
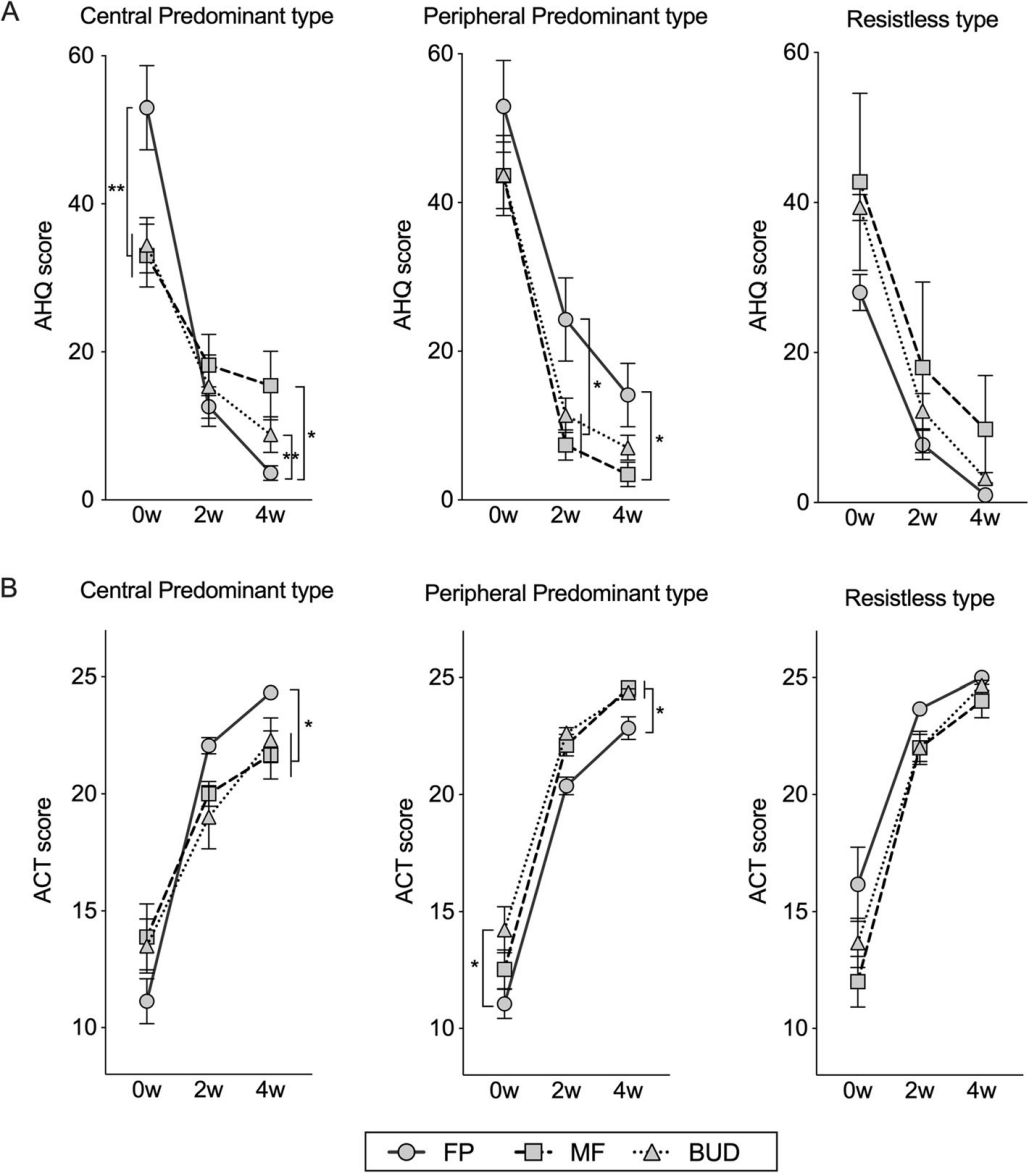
Comparison of the Asthma Health Questionnaire score (**a**) and asthma control test results (**b**) among the ICS therapy groups in terms of subtypes at baseline and after treatment. ICS: inhaled corticosteroid; FP: fluticasone propionate; MF: mometasone furoate; and BUD: budesonide. The bars were expressed as mean ± standard error of mean. The differences among the ICS group were analyzed using the non-repeated analysis of variance with the Student–Newman–Keuls test. *: *p* < 0.05, **: *p* < 0.01

The severity of asthma symptoms and patient-related outcome were evaluated with the AHQ, which is a simple and objective measurement tool for asthma symptoms (Fig. 2a). In the CP type, the FP group had a higher AHQ score than the MF and BUD groups at baseline. The AHQ score did not significantly differ among the three ICS groups in the CP type at 2 weeks after treatment and in the PP type at baseline. By contrast, there were significant differences among the subtypes. In the CP type, the AHQ score of the MF group was significantly higher than that of the FP group (15.4 vs. 3.6, *p* < 0.01) and BUD group (15.4 vs. 8.8, *p* < 0.05) at 4 weeks. In the PP type, the AHQ score of the FP group was significantly higher than that of the MF group (24.3 vs. 7.4, *p* < 0.05) and BUD group (24.3 vs. 11.4, *p* < 0.05) at 2 weeks and the MF group (14.1 vs. 3.4, *p* < 0.05) at 4 weeks.

### Therapeutic evaluation with ACT

Next, we analyzed the ACT, which is a widely used self-administered assessment tool for determining how well a patient’s asthma is controlled. Although there was no statistically significant difference in ACT scores among the three ICP groups in the CP type at baseline, major differences were observed after treatment. The ACT score of the FP group was significantly higher than that of the MF and BUD groups (24.3 vs. 21.7, 22.3, respectively, *p* < 0.05) at 4 weeks after treatment. By contrast, in the PP type, the ACT score of the FP group was lower than that of the MF and BUD groups (22.8 vs. 24.6, 24.4, respectively, *p* < 0.01) at 4 weeks after treatment (Fig. 2b).

### Therapeutic effects on pulmonary function and IOS

We examined whether the classification using the IOS value was correlated to the clinical data before and after treatment. FeNO is useful for the diagnosis and identification of treatment effect in asthma. In this analysis, FeNO improved with treatment. However, no difference was observed between the three groups. Spirometry was an objective parameter relatively similar to IOS, and it had a similar treatment behavior to IOS, but the difference between the three groups after treatment was not significant in FEV1 and FVC. The IOS value itself significantly improved with treatment in all groups, but unlike the respiratory function test values, the difference between the three groups was still observed even after treatment, indicating that IOS may be more effective in reflecting airway impairment (Table 3). When the IOS and respiratory function test results of the participants were compared (Table 4), most items were found to be correlated. However, no significant association was observed between most spirometric value and R20, which is an indicator of the central airway function, and only %PEF was correlated to the IOS value.

**Table 3:** Comparison between the impulse oscillometry system and spirometry in terms of subtypes at baseline (pre) and after treatment (post)

		All	Subtypes			P	Post-hoc test		
			CP	PP	R		CP-PP	CP-R	PP-R
**FeNO (ppm)**									
	Pre	70.6	54.9	72.4	93.1	NS			
	Post	28.8	27.4	26.7	31.9	NS			
		**	*	**	**				
**Impulse oscillometry**									
R5 (kPa/L/s)	Pre	0.46	0.44	0.53	0.25	**	**	**	**
	Post	0.31	0.33	0.33	0.22	**	NS	**	**
		**	**	**	NS				
R20	Pre	0.31	0.37	0.30	0.22	**	**	**	**
	Post	0.26	0.30	0.25	0.19	**	**	**	**
		**	**	**	NS				
R5-R20	Pre	0.15	0.07	0.23	0.03	**	**	NS	**
	Post	0.06	0.04	0.08	0.02	**	**	NS	**
		**	NS	**	NS				
X5	Pre	−0.24	−0.16	−0.33	−0.12	**	**	NS	**
	Post	−0.12	−0.12	−0.13	−0.09	*	NS	NS	*
		**	**	**	**				
Fres	Pre	19.8	15.7	24.0	14.0	**	**	NS	**
	Post	14.2	12.2	15.9	11.4	**	*	NS	**
		**	**	**	*				
AX	Pre	1.84	0.81	2.91	0.39	**	**	NS	**
	Post	0.54	0.38	0.69	0.24	**	*	NS	**
		**	**	**	*				
**Spirometric values**									
%FVC	Pre	80.9	82.6	77.0	91.0	*	NS	NS	**
	Post	99.0	97.8	99.2	100.4	NS			
		**	**	**	*				
%FEV1	Pre	67.5	72.4	61.4	77.9	**	*	NS	**
	Post	92.7	94.2	91.2	95.2	NS			
		**	**	**	**				
FEV1/FVC ratio	Pre	69.6	75.1	65.2	72.8	**	**	NS	*
	Post	78.9	81.9	77.0	80.3	*	*	NS	NS
		**	**	**	**				
%MMEF	Pre	34.0	43.8	26.1	41.3	**	**	NS	**
	Post	61.0	67.9	55.5	68.2	*	NS	NS	NS
		**	**	**	**				
%PEF	Pre	73.1	74.2	66.4	95.3	**	NS	**	**
	Post	103.9	100.9	102.4	115.3	*	NS	*	**
		**	**	**	**				

Data were presented as mean (standard error of mean) or number (percentage). The differences among the subtypes were evaluated using non-repeated analysis of variance (†P) with the Student–Newman–Keuls test. *: *p* < 0.05, **: *p* < 0.01 and NS: not significant. CP-PP: between the central predominant type and peripheral predominant type; CP-R: between the central predominant type and resistless type; and PP-R: between the peripheral predominant type and resistless type

**Table 4:** Correlation between impulse oscillometry system and spirometric parameters without grouping

		%FVC	%FEV1	FEV1/FVC ratio	%MMEF	%PEF
R5	r	0.3	0.42	0.46	0.45	0.54
	P	0.0013**	5.3E–06**	6.9E–07**	7.8E–07**	2.3E–09**
R20	r	0.02	0.06	0.07	0.11	0.27
	P	0.85	0.54	0.44	0.24	0.005**
R5 − R20	r	0.36	0.52	0.58	0.54	0.52
	P	0.0005**	1.2E–08**	9.0E–09**	2.9E–09**	6.9E–09**
X5	r	0.51	0.62	0.61	0.54	0.64
	P	2.3E–08 **	9.9E–13**	1.2E–12**	1.8E–09**	9.0E–14**
Fres	r	0.45	0.61	0.65	0.6	0.55
	P	8.6E–07**	2.2E–12**	4.4E–14**	4.1E–10**	6.7E–10**
AX	r	0.47	0.61	0.62	0.55	0.61
	P	2.2E–07**	4.1E–12**	5.7E–13**	6.5E–10**	3.3E–12**

Data were presented as the Pearson correlation coefficient (r) and *p*-value (P) via a linear regression analysis. **: *p* < 0.01

## Discussion

The current study showed that the differences in asthma phenotypes based on the IOS parameters might be correlated to the efficacy of ICSs. Moreover, IOS was significantly correlated to the severity of asthma. Our previous study has revealed that the phenotypic differences in IOS parameters were associated with the efficacy of ICSs in CVA patients [11]. Thus, we examined the role of IOS in BA in this study. Based on the observation of IOS, it became clear that the phenotypes of BA predominating in central airway lesions and BA predominating in peripheral airway lesions exist. According to the result, the participants were divided based on three subtypes, which were as follows: the CP, PP, and R types. In terms of background characteristics, the participants did not significantly differ (Table 2). However, the age of the participants with the CP type was lower than that of participants with the PP and R type. This result may reflect that age at onset in the CP type was earlier than that in the PP type. The spirometry values differed at baseline in the three groups. The %FEV1, FEV1/FVC, and %MMEF of the PP type were lower than those of the CP and R types (Table 2). Some studies showed that the small airways were the major site of inflammation and obstruction in asthma [26–31]. Thus, we believed that the PP type may be more severe than the CP and R types. Moreover, IOS is a useful device used for the simultaneous assessment of diseases in the peripheral and large airways. Therefore, the role of IOS in BA therapy was examined in this study, which first reported about the subtype of bronchial asthma using IOS. The efficacy of FP and BUD was highest in the CP type. In the PP type, the AHQ score decreased in the MF group compared with FP group (Fig. 2a). Regarding the ACT in the CP type, the score of the FP group was significantly higher than that of the MF group. In the PP type, the ACT score of the FP group was significantly lower than that of the MF group (Fig. 2b). These results indicate that ICSs with coarse- and fine particles are most suitable for the CP and PP type, respectively. Considering these results, the differences in phenotypes based on the IOS parameters might be correlated to the efficacy of ICSs. Thus, IOS examination should be performed on patients with BA prior to ICS administration, and it is better to use ICS with a coarse-particle size for the CP type and ICS with a fine-particle size for the PP type.

The deposition of different sized particles in central versus peripheral airways is controversial. Baou EL reported that in a meta-analysis of studies on patients with asthma, no significant differences were observed between smaller size and larger standard size particle of ICS for change in spirometry. Although there was a relationship between smaller particle size and increased delivery to the distal lung, increased deposition in the distal lung did not improve the clinical outcomes in patients with asthma [32]. On the other hand, this study indicated that there may be a certain relationship between the site of airway impairment and the particle size of ICS. In considering the impact of ICS with different particle sizes, we believe that it could be important to understand the factors influencing the pattern of deposition of an aerosol in the airway and alveolar compartment. The existing tools used to evaluate central and peripheral airway function such as sputum induction (early or late phase sputum), high-resolution computed tomography (airway thickness or air trapping), and exhaled nitric oxide (bronchial or alveolar eNO) are not easier methods in outpatient assessment of peripheral and central airway parameters than IOS. Therefore, we consider IOS to be a useful tool in the treatment of asthma.

When we reconsidered the patient group using the IOS values, we focused on a group called the whole airway type, which was characterized by high R20 and high R5 − R20 (Table S2). This group had the lowest spirometry value, the highest AHQ score, and the lowest ACT score. The whole airway type is believed to present with inflammation or remodeling in both the central and peripheral airways. As previously mentioned, coarse-particle ICSs are suitable for CP type and fine-particle ICSs for PP type. Thus, if a patient with refractory asthma have high R20 and R5 − R20, combination therapy with coarse- and fine-particle ICSs should be considered.

The current study had some limitations. Since IOS is an emerging technique, data about the exact meaning, interpretation, and clinical application of IOS parameters versus spirometric parameters are limited. The reference values used in this study are based primarily on data obtained from Caucasians, and they might cause bias when adapted in Japanese. Asthma that is refractory to ICS will be excluded, but it is difficult to include it in this study because such cases have not been diagnosed with asthma. Several ICSs are randomly prescribed by multiple physicians in their routine asthma treatment practice. However, some bias might exist in the prescription of ICS. Furthermore, the follow-up period in the current study was short. This study was a retrospective observational research assuming no difference between salmeterol and formoterol, but the results must be confirmed in a prospective study, and moreover, the aerosol types of ICS were not examined. Thus, further research must be conducted to identify the most effective individualized treatment for BA using IOS.

## Conclusions

The effect of treatment with ICSs and their correlation to the parameters of IOS in patients with BA were investigated. The phenotypic differences in IOS parameters might be associated with the efficacy of ICSs. That is, coarse-particle ICSs were found to be effective for patients with central airway resistance and fine-particle ICSs for those with peripheral airway resistance. In addition, IOS could detect the diversity in airway dysfunction, thereby indicating its efficacy considering that the lesion sites are the central and peripheral airways. Hence, we recommend the use of IOS in the examination of BA before ICS administration. Moreover, IOS was effective when used in the selection of ICS and the evaluation of the BA phenotype.

## Supplementary information

**Additional file 1: Table S1.** Comparison of characteristics among ICS groups in BA subtypes at baseline. Data were presented as mean (standard error of mean) or number (percentage). Definition of abbreviations are same as indicated in Table 2. **Table S2.** Data were presented as mean (standard error of mean). Differences between the whole type and other type were analyzed by a paired t-test (†P). *: *p* < .05, **: *p* < .01 and NS: no significant.

## Data Availability

Please contact author for data requests.

## Abbreviations

IOS: Impulse oscillometry system
AHQ: Asthma Health Questionnaire
ACT: Asthma control test
ICS: Inhaled corticosteroid
FVC: Forced vital capacity
FEV1: Forced expiratory volume in 1 s
MMEF: Maximum mid-expiratory flow
PEF: Peak expiratory flow
R5: Resistance at 5 Hz
R20: Resistance at 20 Hz
R5 − R20: Resistance at 5–20 Hz
X5: Reactance at 5 Hz
Fres: Resonance frequency
AX: Area of low-frequency reactance
