# Supplementary material for: A retrospective analysis of usefulness of impulse oscillometry system in the treatment of asthma
Source: Respir Res. 2020 Aug 31;21:226. doi: 10.1186/s12931-020-01494-x (PMC7457354; doi:10.1186/s12931-020-01494-x)
Supplement: Supplementary file 1 — Additional file 1: Table S1. Comparison of characteristics among ICS groups in BA subtypes at baseline. Data were presented as mean (standard error of mean) or number (percentage). Definition of abbreviations are same as indicated in Table 2. Table S2. Data were presented as mean (standard error of mean). Differences between the whole type and other type were analyzed by a paired t-test (†P). *: p < .05, **: p < .01 and NS: no significant. [file 12931_2020_1494_MOESM1_ESM.docx]

**ADDITIONAL FILE**

**Real-world efficacy of the impulse oscillometry system for the management of bronchial asthma**

Hiroyuki Sugawara, Atsushi Saito, Saori Yokoyama, Kazunori Tsunematsu,

Hirofumi Chiba, and Hiroki Takahashi

**Table S1.**

**Table S2.**

**RESULTS**

**Table S1**. Comparison of characteristics among ICS groups in BA subtypes at baseline. Data were presented as mean (standard error of mean) or number (percentage). Definition of abbreviations are same as indicated in Table 2.

|  | Central predominant type | | |  | Peripheral predomiant type | | |  | Restless type | | |  |
| --- | --- | --- | --- | --- | --- | --- | --- | --- | --- | --- | --- | --- |
|  | FP | MF | BUD | P | FP | MF | BUD | P | FP | MF | BUD | P |
| n | 15 | 9 | 10 |  | 19 | 17 | 22 |  | 6 | 4 | 6 |  |
| Age | 44.2 (4.4) | 36.7 (4.9) | 41.2 (84.9) | 0.55 | 48.1 (4.5) | 57.4(4.1) | 51.2 (3.5) | 0.28 | 48.5 (8.5) | 48.3 (8.1) | 52.7 (5.3) | 0.97 |
| Male/Female | 6 / 9 | 4 / 5 | 4 / 6 | NS | 7 / 12 | 5 / 12 | 7 / 15 | NS | 5 / 1 | 3 / 1 | 3 / 3 | NS |
| Duration of disease (years) | 4.3 (2.1) | 3.5 (1.5) | 7.9 (3.1) | 0.49 | 6.8 (1.8) | 4.4 (1.8) | 5.0 (1.2) | 0.55 | 2.5 (1.5) | 2.1 (1.7) | 2.5 (0.8) | 0.97 |
| Smoker/non-smoker | 8 / 7 | 6 / 3 | 5 / 5 | NS | 14 / 5 | 10 / 7 | 13 / 9 | NS | 5 / 1 | 3 / 1 | 4 / 2 | NS |
| BMI | 23.4 (1.2) | 27.5 (1.8) | 27.2 (1.6) | 0.09 | 26.6 (1.3) | 25.0 (1.1) | 25.2 (10) | 0.55 | 22.8 (1.1) | 23.6 (3.3) | 24.9 (1.7) | 0.31 |
| Total IgE level (IU/mL) | 565.0 (207.7) | 213.6 (75.2) | 435.5 (354.2) | 0.2 | 430.3 (121.6) | 331.5 (117.0) | 405.6 (158.4) | 0.5 | 580.0 (151.1) | 442.3 (217.8) | 263.0 (93.0) | 0.31 |
| Eosinophil in blood (/μl) | 221.1 (46.7) | 215.0 (69.5) | 249.3 (134.4) | 0.77 | 315.8 (41.0) | 262.1 (37.2) | 333.7 (37.6) | 0.4 | 238.5 (54.2) | 296.5 (52.9) | 286.7 (77.5) | 0.8 |
| FeNO (ppb) | 78.4 (18.0) | 36.0 (13.8) | 36.5 (10.1) | 0.1 | 63.8 (11.0) | 85.2 (17.7) | 70.0 (13.4) | 0.57 | 136.0 (10.6) | 73.3 (19.1) | 81.8 (26.0) | 0.09 |

**Table S2.** Data were presented as mean (standard error of mean). Differences between the whole type and other type were analyzed by a paired t-test (†P). *: p<.05, **: p<.01 and NS: no significant.

|  | subtype | |  |  |
| --- | --- | --- | --- | --- |
|  | Whole type | Others | P |  |
| n | 36 | 72 |  |  |
| Spirometric values |  |  |  |  |
| % FVC | 76.8 (3.4) | 83.0 (1.9) | 0.08 | NS |
| % FEV1 | 57.6 (3.0) | 72.4 (2.2) | 0.00015 | ** |
| FEV1/FVC ratio | 63.7 (1.7) | 72,5 (1.3) | 0.0001 | ** |
| %MMEF | 23.8 (2.0) | 39.2 (2.6) | 0.00014 | ** |
| % PEF | 61.1 (3.7) | 79.2 (2.9) | 0.00027 | ** |
| AHQ score | 50.2 (3.3) | 40.4 (4.4) | 0.023 | * |
| ACT score | 12.3 (0.7) | 13.2 (0.8) | 0.23 | NS |
